# Diesel exhaust: current knowledge of adverse effects and underlying cellular mechanisms

**DOI:** 10.1007/s00204-016-1736-5

**Published:** 2016-05-10

**Authors:** Sandro Steiner, Christoph Bisig, Alke Petri-Fink, Barbara Rothen-Rutishauser

**Affiliations:** Adolphe Merkle Institute, University of Fribourg, Chemin des Verdiers 4, 1700 Fribourg, Switzerland

**Keywords:** Diesel engines, Diesel particles, Ultrafine particles, Lung cells, Adverse effects

## Abstract

Diesel engine emissions are among the most prevalent anthropogenic pollutants worldwide, and with the growing popularity of diesel-fueled engines in the private transportation sector, they are becoming increasingly widespread in densely populated urban regions. However, a large number of toxicological studies clearly show that diesel engine emissions profoundly affect human health. Thus the interest in the molecular and cellular mechanisms underlying these effects is large, especially concerning the nature of the components of diesel exhaust responsible for the effects and how they could be eliminated from the exhaust. This review describes the fundamental properties of diesel exhaust as well as the human respiratory tract and concludes that adverse health effects of diesel exhaust not only emerge from its chemical composition, but also from the interplay between its physical properties, the physiological and cellular properties, and function of the human respiratory tract. Furthermore, the primary molecular and cellular mechanisms triggered by diesel exhaust exposure, as well as the fundamentals of the methods for toxicological testing of diesel exhaust toxicity, are described. The key aspects of adverse effects induced by diesel exhaust exposure described herein will be important for regulators to support or ban certain technologies or to legitimate incentives for the development of promising new technologies such as catalytic diesel particle filters.

## The diesel engine: opportunity and challenge

In the year 312 BC, the Roman censor Appius Claudius Caecus built the Via Appia, a paved road connecting Rome and Capua. This was the beginning of a process that culminated in a monumental network of around 80,000 km of paved roads connecting the cities of the Roman Empire, which allowed efficient transportation of goods, exchange of information, and movement of troops. The Romans knew very well that cultural and economic development, security and safe supply of food and resources—essentially the concept of a state—rises and falls with the degree of provided mobility.

More than 2000 years later this rule still applies, perhaps even more than ever before, in the globalized world we live in. It is to a large part the invention of thermal engines—first the steam engine in the eighteenth century, then the internal combustion engines in the nineteenth century—that made globalization possible, and the economic, social and governmental structures we live in cannot be imagined without engine-driven industry and transportation.

Despite all the great advantages, the downside of internal combustion engines is evident: Their ubiquitousness has created one of the major challenges for society and the environment—their emissions affect human health on a regional scale, and the earth’s climate even on a global scale. Strikingly, this only began to be fully acknowledged during and after the 1960s, when first attempts to regulate exhaust emissions were undertaken, for instance by the Environmental Protection Agency of the United States (http://www3.epa.gov).

Of all existing internal combustion engines, the diesel engine is among the most popular and is therefore of great concern with respect to the environment and public health. After its invention in the late 1890s, the diesel engine rapidly evolved into a prominent technology for heavy-duty applications in the stationary, marine, railroad and military sectors, but at first did not feature heavily in the private transportation sector. Only in the late 1980s, with the adaptation of turbochargers and the development of common rail direct injection systems, were diesel engines made feasible for the light-duty, private transportation sector. Since then, their popularity has continuously increased, to a large part certainly due to obvious reasons such as their higher durability and higher fuel efficiency compared to gasoline cars. As discussed in detail by Cames and Helmers (2013), there are further, less obvious reasons for their increasing popularity. Most notably, governmental market interventions had (and still have) a strong influence, and it is therefore unsurprising that the trend is not globally uniform but depends on the economic and ecological strategies pursued by different governments. Among the largest car markets, the European Union and India show high and rapidly growing shares of diesel cars, whereas in the USA, China and Japan and Brazil, they are rather a side issue.

With diesel engines penetrating the private transportation sector, their emissions are brought into urban centers, inevitably resulting in a high and continuous exposure of a large part of the population. In 2010, 95 % of the urban atmospheric background mass concentrations of particulate elemental carbon, and between 10 and 35 % of the atmospheric organic carbon mass in the ambient air of Birmingham (UK), could be attributed to diesel engines (Yin et al. 2010). In Delhi, India, many studies have source-appointed PM and found up to 28 percent of total PM originating from diesel vehicles (Pant and Harrison 2012). It is figures like these that bring the health effects of diesel engine emissions into focus, particularly since in many regions of the world (e.g., India) adequate emission legislations are introduced with a significant time lag compared to more developed countries. Not surprisingly in an era of strong scientific, economic and regulatory interest in public health, diesel engine emissions happen to be among the most thoroughly investigated anthropogenic pollutants. This is reflected in the large number of scientific publications in this field, of which only an impression can be given here (Varatharajan and Cheralathan 2012; Brijesh and Sreedhara 2013; Dwivedi et al. 2013; Giakoumis et al. 2013; Myung and Park 2012; Geller et al. 2006; Wierzbicka et al. 2014; Heeb et al. 2013; Heeb et al. 2010; Ashraful et al. 2015; Popovicheva et al. 2015; Imtenan et al. 2014).

The toxicity of any inhaled environmental agent is the result of its interaction with the respiratory tract, which is in constant contact with the air. The resulting toxicity is thus the result of the agent’s properties on one hand, and of the properties or working principle of the respiratory tract on the other. Before giving an overview of the molecular and cellular mechanisms by which diesel engine emissions affect human health, we will give a brief overview on the composition and physicochemical characteristics of diesel exhaust, as well as on the human respiratory tract and its functional properties that are most relevant to the inhalation of air pollutants and the attributed health effects.

## Diesel exhaust composition

**Fig. 1:**
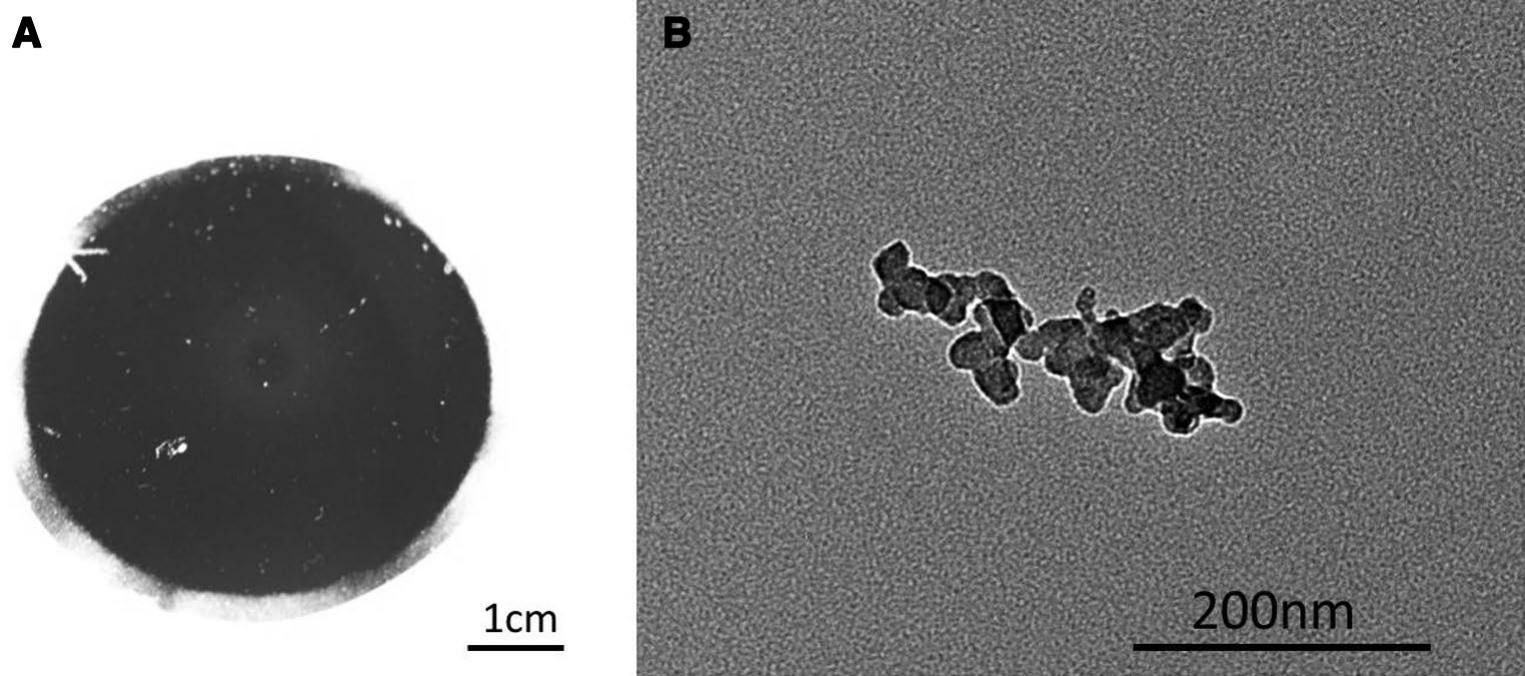
Diesel particles collected on a filter from an unfiltered diesel passenger car, driven for 30 min, resulting in approximately 5 mg of particles (**a**) and transmission electron micrograph of diesel exhaust particles (**b**). The picture shows representative irregularly shaped elongated particle aggregates of ca. 200 nm length. The primary particles of about 20 nm are clearly identifiable

Diesel exhaust is a complex mixture, but is made up of three fractions: solid, condensed (or liquid), and gaseous fractions (Chan et al. 2007; Westerholm and Egeback 1994; Jayaram et al. 2011). The solid fraction mainly consists of primary particles with a core of about 10–30 nm in diameter composed of elemental carbon present in a graphitized form (Zhu et al. 2005; Liati and Eggenschwiler 2010). The primary particles can then agglomerate and form larger soot aggregates with mean diameters of 60–100 nm (Burtscher 2005; van Setten et al. 2001), which are the main cause of the black appearance of unfiltered diesel exhaust (Fig. 1). The exact mechanisms underlying the formation of soot are complex and only partly understood. The main pathway appears to be pyrolyzation of unburnt fuel and lubrication oil, mainly to ethyne, followed by polymerization reactions, ring closure and stacking of the resulting polycyclic, graphite-like sheets in a turbostrated structure. In addition to the elemental carbon in the soot particles, the solid fraction of diesel exhaust contains metal and metal-oxides originating from lubrication and fuel additives and from engine wear. Fuel and lubrication oil additives contain metals as functional components, such as zinc, and magnesium in oils and cerium, iron, manganese, platinum and copper in fuels (Mayer et al. 2010). At the high temperatures present in the combustion chamber, these additives may be subject to vaporization, condensation and nucleation processes, resulting in their embedding into the carbonaceous soot particles and in the formation of metal and/or metal-oxide nucleation mode particles with diameters ranging down to 10 nm (Burtscher 2005; Mayer et al. 2010; Maricq 2007). In addition, wear on the engine, which is predominantly made up of iron (Okuda et al. 2009), produces particles in the size range of 1–2 µm and higher, which may be subject to the same processes as the metals in fuel and lubrication oil additives and may ultimately also participate in the formation of nanosized particles and the enrichment of the carbonaceous soot particles with iron and iron oxides (Mayer et al. 2010).

However, by far the most prevalent element to be found in the solid diesel exhaust particle fraction is elemental carbon, which is highly biopersistent. Therefore, whatever chemical compounds these particles contain, they will only to a limited extent dissolve in biofluids such as the lung-lining layer. As a consequence, even though the elemental composition is known to play a certain role in defining the toxicity of the solid particle cores (Steiner et al. 2012; Ma et al. 2014), it is not the main factor. It is instead the toxic compounds that adsorb on particle surfaces, as described later on, and the surface properties of the particles that deserve special attention. During the combustion process, but also later due to the presence of oxidizing gases and/or photochemical processes, the surfaces of the carbon particles become chemically activated, for example by the formation of quinones by the partial oxidation of particle-bound polyaromatic hydrocarbons (PAHs). Once activated, the particles participate in redox reactions, resulting in the formation of reactive oxygen species (ROS) such as hydrogen peroxide. Furthermore, the metals and metal-oxides—present as individual particles or embedded in carbon particles—participate in Fenton-type reactions by which the potency of the ROS is further increased (Geller et al. 2006; Antiñolo et al. 2015; Squadrito et al. 2001; McWhinney et al. 2013).

The gaseous exhaust fraction consists of more than 99 % by volume of non-toxic inorganic gases such as nitrogen, water and oxygen. Toxic inorganic gases such as carbon dioxide (CO_2_), carbon monoxide (CO), nitric oxide (NO) and nitrogen dioxide (NO_2_), and a complex mixture of organic compounds account for the rest. The organic fraction consists of small molecules such as methanol, ethylene or formaldehyde but mainly of larger aliphatic compounds and more complex molecules such as benzene, naphthalene, pyrene, anthracene and their various functionalized derivatives, collectively referred to as PAHs, nitrated polyaromatic hydrocarbons (NPAHs) and heterocyclic aromatic compounds (HACs) (Heeb et al. 2010; Westerholm and Egeback 1994; Vieira de Souza and Corrêa 2015; Heeb et al. 2008; Zielinska et al. 2004). Sources of the organic exhaust components are traces of fuel and lubrication oil that survived combustion, but also compounds that cannot be found in the fuel or oil and are newly formed from partially combusted precursors (Rhead and Hardy 2003).

With increasing molecular weight, and depending on their functionalization and the exhaust temperature, organic compounds may not be present as gases but are adsorbed onto soot, metal and metal-oxide particles or condense to form particles that together with water droplets comprise the liquid exhaust fraction (Burtscher 2005; Zielinska et al. 2004). Hence, for many of the organic exhaust components, a clear attribution to the gaseous or condensed fraction is difficult. Their distribution instead depends on the equilibrium between condensation and evaporation, which in turn is a function of exhaust temperature, exhaust concentration and the availability of nucleation centers and surfaces for condensation in the exhaust (e.g., particles).

The composition of all three exhaust fractions strongly depends on engine type and operation mode (inclusively electronic control and after-treatment systems), fuel and lubrication oil type and fuel and lubrication oil additives (Giakoumis et al. 2013; Popovicheva et al. 2015; Imtenan et al. 2014; Zhu et al. 2005; Mayer et al. 2010; Zielinska et al. 2004; Mathis et al. 2005; McClellan et al. 2012).

Revolutionary developments in exhaust after treatment have been done, and new engines with the best after-treatment systems emit substantially less PM and gaseous compounds (Khalek et al. 2015). Nonetheless, there is still a significant amount of older technology cars on the road, also actions to remove PM with a diesel particulate filter (DPF) usually result in an increase of NOx and vice versa and may form—tough fewer in number—different kinds of particles (McClellan et al. 2012; Khalek et al. 2011).

## The respiratory system

It is important to highlight certain aspects of the lung structure in order to understand how diesel exhaust can interact with lung cells and possible resulting adverse effects.

The respiratory tract provides an enormous internal surface of ca. 150 m^2^ that is optimized for facile exchange of gases between inhaled air and the bloodstream (Gehr et al. 1978). It can broadly be subdivided into the nasopharyngeal, the tracheobronchial and the alveolar regions (Oberdorster et al. 2005). The tracheobronchial region can further be subdivided into the trachea, the bronchi (main, lobar, segmental bronchi and bronchioles), and the alveolar region into alveolar bronchioles and the alveoli. Gas exchange between inhaled air and the bloodstream only takes place in the alveolar bronchioles and the alveoli (Rothen-Rutishauser et al. 2008).

Given the large surface area the respiratory tract represents, the importance of efficient mechanisms for avoiding the entry of airborne pollutants—primarily particulate ones—is evident. A first line of defense, active in the nasopharyngeal and the upper tracheobronchial region, is comprised by sneezing and coughing. Once the lower tracheobronchial region is penetrated, inhaled particles may be trapped in the mucus lining of the airway epithelium. This filtration of particles from the inhaled air is of different efficiency for different particles sizes and in different regions of the respiratory tract (Oberdorster et al. 2005; Carvalho et al. 2011). As a consequence, different regions of the respiratory tract will be exposed to different size ranges of particles: the nasopharyngeal region mainly to particles larger than 500 nm and smaller than 5 nm, the tracheobronchial region mainly to particles in the size range of 1–50 nm, and the alveolar region mainly to particles in the size range of 5–500 nm (Oberdorster et al. 2005; Patton and Byron 2007).

**Fig. 2:**
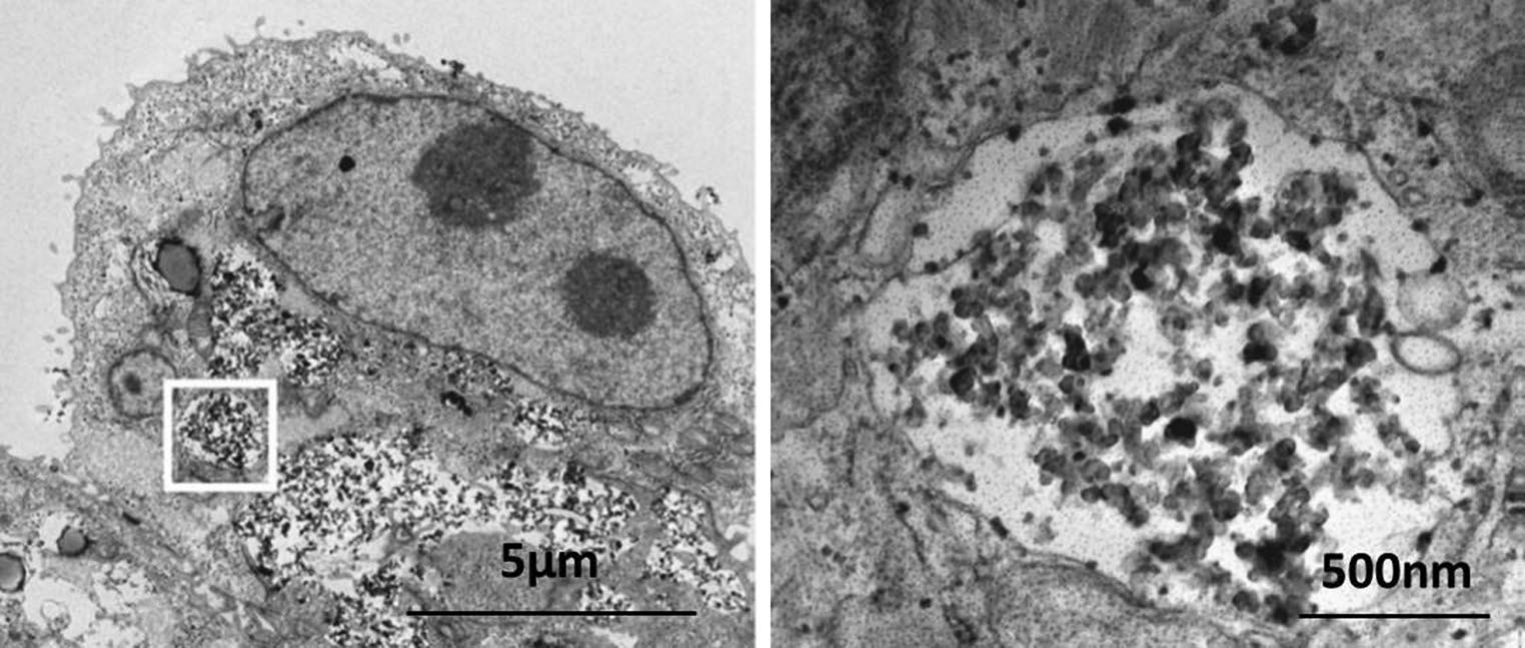
After deposition in the respiratory tract, diesel exhaust particles are normally cleared from the epithelium by the mucociliary escalator or by phagocytosis by resident macrophages. Other cell types may, however, also take up the particles, which due to their biopersistence results in particle accumulation in the tissue and thereby extensive exposure to the redox-active particle surfaces. The figure shows transmission electron micrographs of diesel exhaust particles that were taken up in vitro by bronchial epithelial cells (cell line 16HBE14o–). The picture on the *right* shows a magnified part of the picture on the *left* [adapted from Lehmann et al. (2009)]

Even though a large fraction of airborne particles is filtered from the inhaled air and eliminated via the mucociliary escalator, bronchiolar and alveolar deposition of particles still occurs and the mechanisms responsible for their clearance changes gradually from the nasopharyngeal to the most peripheral (alveolar) region. Clearance of deposited particles from the airways is fast (within minutes) and is mainly achieved by the ciliary movement of the mucus toward the pharynx where it is swallowed together with any deposited material (Kilburn 1968). The mucociliary activity gradually declines from the bronchiolar to the alveolar region and in the alveoli, epithelial clearance fully relies on other mechanisms, mainly on the action of lung-resident professional phagocytes, the alveolar macrophages that can engulf inhaled diesel particles. These cells patrol the luminal side of the lung epithelium and take up any deposited material they encounter and together with pulmonary dendritic cells, collaborate as sentinels against deposited fine particles (Rothen-Rutishauser et al. 2008; Moller et al. 2008; Ochs and Weibel 2008; Vermaelen et al. 2001). This clearance is considerably slower (over weeks or months) than the mucociliary elevator, which allows the particles to interact with the respiratory epithelium for prolonged periods of time (Moller et al. 2008). Besides possible chemical interactions, it has been shown that deposited particles may translocate across the epithelium into the connective tissue, the bloodstream or the lymphatic circulation (Oberdorster et al. 2005) and that they may be taken up by various cell types other than macrophages, for instance epithelial cells of the respiratory tract (Lehmann et al. 2009; Fig. 2). Exactly how this happens is not well understood but appears to involve active and passive processes (Muhlfeld et al. 2008).

Taken together, the nature of the mechanisms for protection and clearance has important consequences for how air pollution interacts with the respiratory system:

1. They only work for particulate air pollution but cannot defend the organism against adverse effects of gaseous compounds. In fact, due to the function of the lungs, gases must be able to freely enter the respiratory system and the respiratory epithelium is specifically designed to allow efficient exchange of gases between the inhaled air and the bloodstream.
2. Airborne particles reach different regions of the respiratory system depending on their size. Smaller particles reach more peripheral regions of the lung than larger particles.

As a consequence, the occurrence of adverse effects of diesel exhaust is not only a function of the overall concentration of many different chemical species, but also of the combined effects of the gaseous, liquid and particulate exhaust fraction and how individual compounds are distributed between them. Since the particle size ultimately determines the major site of deposition, the overall particle number-size distribution as well as the allocation of individual compounds to specific size classes is of importance in addition.

## Mechanisms underlying adverse effects of air pollution

Epidemiological studies conducted over the last two decades have shown a positive correlation between the level of particulate air pollution and increased adverse health effects (Dockery et al. 1993; Bremner et al. 1999; Braga et al. 2000), including increased pulmonary diseases (Choudhury et al. 1997; Pope et al. 1999), as well as a rise in the number of deaths from cardiovascular disease (Abbey et al. 1999; Aga et al. 2003; Zanobetti et al. 2003; Kaiser et al. 2004). Based on the proven genotoxicity of its constituents, diesel exhaust has been judged as mutagenic and carcinogenic to humans by the World Health Organization (WHO 2010, 2010) and in June 2012, the International Agency for Research on Cancer (IARC) classified diesel engine exhaust as a group 1 carcinogen to humans (2013), predominantly based upon epidemiological studies (Attfield et al. 2012; Silverman et al. 2012), supported by a large number of experimental studies (Hemmingsen et al. 2011; Sevastyanova et al. 2008; Topinka et al. 2012; Fall et al. 2007; Risom et al. 2003).

The exact causal connection between air pollution—including diesel exhaust—and adverse health effects is still not fully understood, but certain molecular and cellular mechanisms are generally assumed to play a key role. In the following, these mechanisms will be described with an emphasis on why diesel exhaust—at least in its non-treated form (i.e., generated in absence of filtration or catalytic converters) represents a worst-case scenario for respiratory health.

The most well-described cellular responses upon interaction with diesel exhaust are the induction of pulmonary oxidative stress and (pro-)inflammation, both of which are known to be involved in the onset or exacerbation of respiratory diseases such chronic obstructive pulmonary disease (COPD), but also in the (air pollution-related) development of systemic effects such as cardiovascular diseases or thrombosis. A further relevant reaction is the induction of genotoxicity, which is partly also linked to oxidative stress and (pro-)inflammation and may ultimately result in the onset of lung cancer (Moller et al. 2008; Schwarze et al. 2013). None of the three endpoints are specific to diesel exhaust, but apply for all kinds of air pollution or inhaled agents with adverse health effects (such as tobacco smoke) and will therefore be described in a generalized manner.

**Fig. 3:**
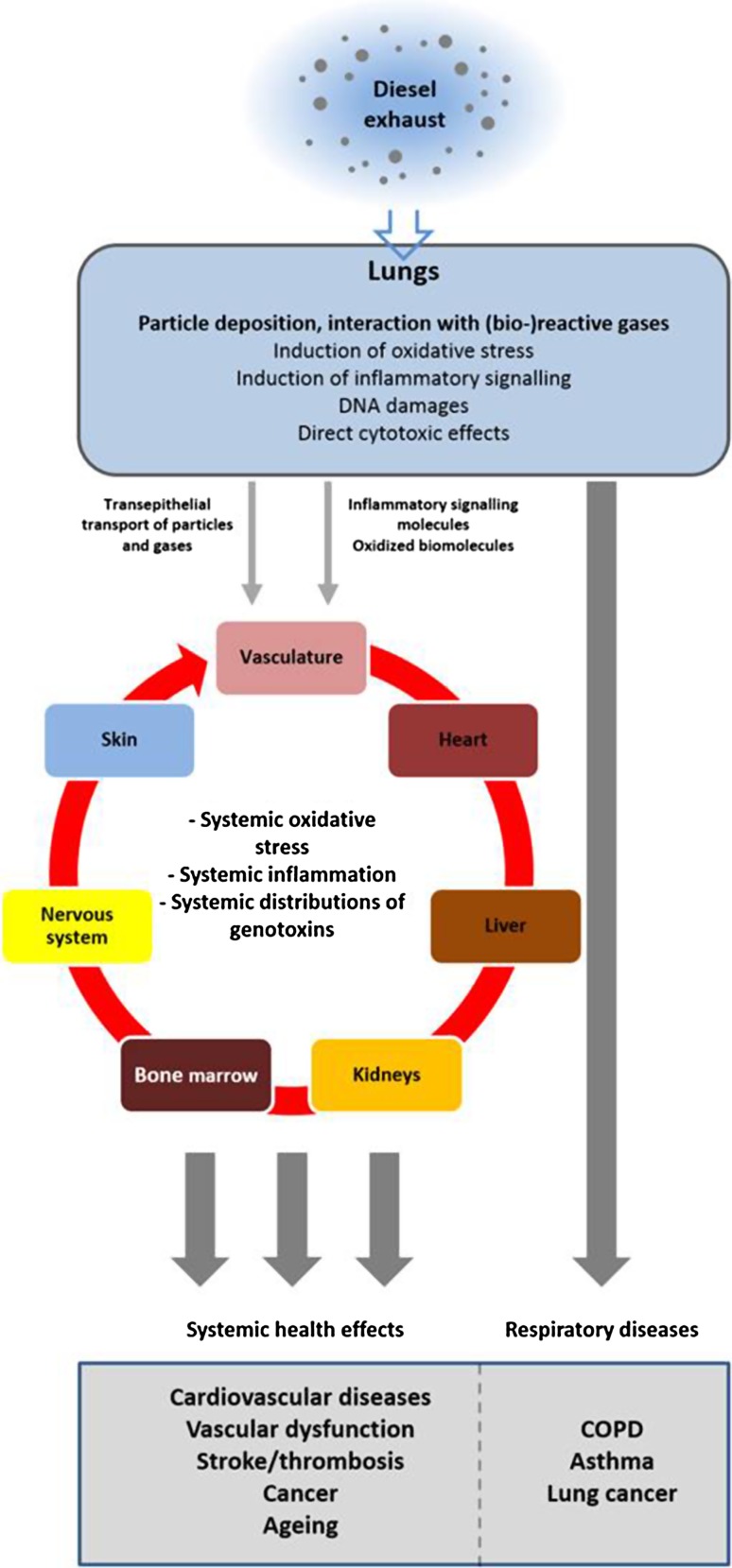
Inhalation of diesel exhaust directly affects the respiratory tract by inducing local oxidative stress, (pro-)inflammatory signaling and genotoxicity, which ultimately may result in respiratory diseases such as COPD, asthma and lung cancer. Exhaust components (particles and gases) are also translocated across airway and respiratory epithelia and enter the circulatory system, along with locally produced (pro-)inflammatory signaling molecules and oxidizing molecules that are either actively produced by lung-resident immune cells or are products of redox reactions triggered by exhaust components. The bloodstream distributes these molecules throughout the whole body, resulting in systemic oxidative stress and inflammation and genotoxic effects in organs other than the lung. The ultimate result may be systemic health effects such as cardiovascular diseases, thrombosis, stroke, cancer or accelerated aging

A detailed description on how pulmonary oxidative stress, (pro)inflammation and genotoxicity result in specific pathologies, for instance Chronic Obstructive Pulmonary Disease (COPD), other fibrotic disorders or lung cancer, is beyond the scope of this article, and we will only provide an overview on the link between air pollution and the three mentioned endpoints. For further reading, comprehensive literature on our current understanding of the occurrence of a wide array of fibrotic respiratory disorders (Bonner 2007) as a consequence of pulmonary oxidative stress, inflammation and genotoxicity, including COPD (Decramer et al. 2008; Yang et al. 2011; Gan et al. 2004; Kersul et al. 2011; Wedzicha and Seemungal 2007) and asthma (Alexis and Carlsten 2014; McCreanor et al. 2007), but also lung cancer (Silverman et al. 2012; Attfield et al. 2012; Silverman et al. 2014) and systemic complications such as cardiovascular diseases (Brito et al. 2010; Miller et al. 2015; Chin 2015; Tong et al. 2014; Haberzettl et al. 2014) and thrombosis (Mills et al. 2007), is available elsewhere (Fig. 3).

### Pulmonary oxidative stress and (pro-)inflammation

Even in 1990s it was recognized that adverse health effects caused by particulate air pollution rely on pulmonary inflammation (Seaton et al. 1995), but the underlying mechanisms were not known at that time. In 1994, Oberdorster et al. (1994) demonstrated that intratracheal instillation of ultrafine titanium dioxide (TiO_2_) particles with average diameters of about 20 nm resulted in significantly stronger inflammatory responses in mice than the identical mass of fine TiO_2_ particles with average diameters of about 250 nm. They realized that this pointed toward the particles’ surface area as relevant factor in particle toxicology, i.e., their chemical composition was not the only important factor. Subsequent research identified the induction of oxidative stress, i.e., the formation of reactive oxygen and nitrogen species (ROS/RNS), as a main factor determining particle toxicity. This finding was in perfect agreement with the fact that particle toxicity increases with the particle surface area, since the formation of ROS and RNS at particle surfaces was known to occur (Donaldson et al. 2002).

A direct connection between inflammatory processes and oxidative stress was subsequently also suggested by Xiao et al. (2003) and could explain effects of particle inhalation (Oberdorster et al. 2005; Schins 2002; Schins and Knaapen 2007; Donaldson et al. 2005). Based on their results, it is assumed that ROS formed at the surfaces of redox cycling particles, depending on the extent of their formation, cause either the activation of antioxidant defense mechanisms (at low levels of oxidative stress), (pro-)inflammatory responses (at intermediate levels of oxidative stress) or exert cytotoxic effects (at high levels of oxidative stress). Importantly, an additional promoter of the impact of the smaller fraction of particles is their retention time in the alveolar region and their ability to translocate across the respiratory epithelium and/or enter cells, where they may accumulate over time. Depending on their solubility and biodegradability, which for instance is extremely low for the carbon particles in diesel exhaust, they may remain there for years and exert adverse effects.

ROS and RNS are not only formed at particle surfaces. Many gases in diesel exhaust are themselves ROS or RNS (for instance ozone and NO_2_), while many hydrocarbons, volatile and dissolved from particle surfaces, may actively participate in redox cycling and/or may act as potent electrophiles. Furthermore, metal ions dissolved from particles may catalyze the formation of ROS (Shinyashiki et al. 2009).

The fact that adverse effects of inhaled pollutants are not restricted to the respiratory system could furthermore be attributed to (pro-)inflammatory cytokines, the mediators of inflammation, being distributed via the bloodstream from the respiratory tract to the whole body, causing systemic inflammation. Also the translocation of particles into the blood stream and the resulting induction of systemic oxidative stress have been shown to be involved (Miller et al. 2015; Haberzettl et al. 2014; Simkhovich et al. 2008; Kunzli and Tager 2005). Translocation of molecular oxidants—either present in the inhaled air or formed in the respiratory tract in response to the inhalation of oxidants—may act in the same way. In addition, once inflammatory reactions are triggered, activated immune cells produce ROS and RNS as a part of the immune response, by which the level of oxidative stress may be increased endogenously and oxidative stress may be induced locally in the respiratory tract or systemically (Weitzman and Gordon 1990; Bogdan et al. 2000).

### Oxidative-stress-independent induction of pulmonary inflammation

While the above-described model assumes oxidative stress to be the trigger for the induction of pulmonary inflammation, more recent studies have shown that PAHs may initiate (pro-)inflammatory reactions but do not form ROS or RNS on their own (Podechard et al. 2008; Tsuji et al. 2011). This was found to rely on the activation of (pro-)inflammatory signaling cascades via highly specific interactions between certain PAHs and the intracellular aryl hydrocarbon receptor (AhR) present in many cell types. Interestingly, activation of the AhR signaling pathway results in the intracellular formation of ROS (Nebert et al. 2004), which play an active role as secondary messengers, i.e., they regulate cellular events downstream from the AhR. One of these events is the activation of nuclear factor kappaB (NFκB), one of the key inducers of inflammatory responses (Kalthoff et al. 2010; Stockinger et al. 2011); and indeed it was shown that when cells are treated with antioxidant agents in addition to PAHs, the pro-inflammatory responses are inhibited (Podechard et al. 2008). (Pro-)inflammatory stimulation by complex aerosols is therefore likely to be the result of the combined action of particle-mediated ROS formation and ROS-independent mechanisms.

### Pulmonary genotoxicity

Cancer, including lung cancer, may arise as a result of genotoxicity, the introduction of damages, or changes in the genomic DNA of cells.

An important mechanism underlying genotoxicity relies on oxidative and alkylating DNA damages, which are introduced by organic radicals, ROS and RNS (inhaled or endogenously formed by immune cells or the cellular metabolism), that attack double bonds of purine or pyrimidine bases or cause DNA strand breaks (Reuter et al. 2010; Xue and Warshawsky 2005). The direct action of ROS/RNS as well as intercalation and adduct formation by aromatic organic compounds is referred to as primary genotoxicity, whereas the action of ROS/RNS produced by immune cells in response to (pro-)inflammatory stimulation is classified as secondary genotoxicity (Schins 2002; Schins and Knaapen 2007).

Electrophilic breakage of double bonds results in the formation of adduct radicals which react further to various products that ultimately may cause DNA polymerases to misread the nucleotide sequence during DNA replication and may hence result in the incorporation of the wrong bases (Donaldson et al. 2005; Shinyashiki et al. 2009; Xue and Warshawsky 2005; Cooke et al. 2003; Kasai 2002). Biomolecules other than DNA may be oxidized as well. For instance, peroxidation of polyunsaturated fatty acids results in the formation of reactive aldehydes such as acrolein or crotonaldehyde, which may react with DNA and result in the formation of exocyclic DNA adducts (Nair et al. 2007).

Furthermore, the activity of PAHs, NPAH, and HACs may cause genotoxicity in a way that is less dependent on the presence of oxidizing agents or even oxidative stress. These molecules may directly interact with DNA by intercalation between the stacked bases, DNA adduct formation and/or the formation of abasic sites (Xue and Warshawsky 2005; Ferguson and Denny 2007). The ultimate result is the loss of bases or the incorporation of additional or wrong bases during DNA replication.

## Methods to assess exhaust toxicity

With the known adverse effects caused by the emissions of internal combustion engines toward human health and the environment—and the resulting exhaust emission legislation of increasing stringency—new or optimized technologies are being developed almost on a daily basis. These technologies include highly efficient exhaust after-treatment systems (e.g., diesel particle filters, selective catalytic converters), new engine technologies providing optimized combustion, but also new fuel types (e.g., biodiesel), all of which aim at reducing the net mass emissions of particles, CO, CO_2_, NO, NO_2_, and organic compounds. All of these may result in profound changes in the exhaust composition and whereas the overall reduction in mass emission brought about by new technologies can be measured with relative ease, it is much more difficult to describe how they affect exhaust toxicity, which is clearly the ultimate objective behind exhaust emission legislation.

The basic mechanisms underlying adverse health effects described above provide a valuable (but not exhaustive) set of markers based on which exhaust toxicity can be assessed, i.e., the quantification of (pro-)inflammatory molecules, oxidized biomolecules or DNA damages upon a test system (such as an animal, a cell culture or a cell-free solution of, for instance, DNA) exposed to an exhaust sample. Biochemical methods for assessing these markers (and many others not listed in this review) have been developed, validated and standardized over the past few decades; the bioanalytical aspect is therefore not the greatest challenge when measuring exhaust toxicity.

As is the case for any toxicological experiment, experiments for exhaust toxicity assessment should be designed in a way that they (1) simulate real-world exposure conditions as closely as possible, (2) provide a high level of reproducibility, and (3) are representative for the species of interest, in most cases humans. Simulations of real-world conditions depend on how a test sample of the substance under investigation is produced or collected, and how and at which dose it is administered to the biological test system. The experimental reproducibility and the relevance of the experiment with respect to the species of interest are to a large degree determined by the choice of the test system and the endpoints taken into account.

The high complexity of diesel exhaust, particularly the presence of the dynamically interacting gaseous and particulate fractions, renders adequate sample preparation highly challenging. In order to simulate real-world conditions, it is obviously best performed by sampling exhaust from a test vehicle operated on-road. The exhaust samples should be diluted in a way that does not influence the exhaust particle number-size distribution (Fujitani et al. 2009). Diluted exhaust samples can then either directly be used for exposure experiments or treated in a manner reflecting processes of photo- and atmospheric chemistry that take place under real-world conditions and result in the formation of secondary organic aerosols (SOA) not present in the original exhaust (Zhang et al. 2014; Robinson et al. 2007; Jathar et al. 2014; Atkinson and Arey 1994).

Such close simulation of real-world conditions is, however, difficult to achieve and in many cases is unfeasible. For example, on-road operation of the test vehicle will decrease the experimental reproducibility. In practice, the test vehicle is therefore usually operated on a dynamometer, either under steady-state conditions or running a standardized test cycle. Real-world simulation of the exhaust dilution will decrease exhaust concentrations to such an extent [roughly 1:10,000 (Zhang and Wexler 2004)] that exposure experiments would have to be conducted for very long periods of time in order to obtain detectable effects. Studies performed with complete exhaust (in many studies, sampled exhaust particles or extracts thereof are used instead) therefore usually work with final particle concentrations of 100–300 µg/m^3^, which is considerably higher than the real-world concentrations where the daily average of Particulate Matter 10 (PM_10_) should not exceed 20–25 µg/m^3^ (depending on the country) (Hesterberg et al. 2009; Paur et al. 2011). Photo- and atmospheric chemistry is usually neglected, unless a study specifically investigates such effects.

Experimental reproducibility is influenced by the sample preparation, the exposure method and the biological test system. As long as exhaust samples are produced on a dynamometer, considerable variation between individual samples is unlikely. The same applies for the exposure method, which can be standardized to a high degree. The choice of the biological test system therefore deserves special attention, also with regard to its relevance.

Basically, three different choices are possible: epidemiological studies (the biological system is a specific cohort population), in vivo (where the biological system is an animal or a human) and in vitro studies (where the biological system is a cell or bacterial culture). Epidemiological studies, while certainly providing the most relevant results with respect to public health, are not feasible for the assessment of exhaust toxicity of a specific engine technology, fuel or exhaust after-treatment system The effects of a new technology should be investigated prior to its widespread use; however, epidemiological studies can only give retrospective estimates, i.e., only technologies that already are in widespread use can cause effects that may be detected in this way.

In vivo studies offer the advantage of testing on complete living organisms that comprise a whole regulatory network and are able to display all relevant responses to a given treatment. This renders the experimental relevance high, particularly when studies are performed with humans as test systems. However, human exposures, even though frequently performed (Hesterberg et al. 2009, 2010; Ghio et al. 2012) suffer from limited sample accessibility after exposure, which restricts the choice of biological endpoints. Also, such studies covering accumulative effects over the lifetime of an individual are practically unfeasible. In contrast, animal studies allow using the whole body as sample for biological investigation and because of the short lifetime of most model animals (for instance mice or rats), studies covering all developmental stages can be performed (e.g., the health effects institutes (HEI) exposures of new technology diesel exhaust (McDonald et al. 2015). These studies, however, suffer from a lack of comparability to humans with respect to the required doses and the resulting responses, both of which cannot necessarily be extrapolated between different species (Hesterberg et al. 2009; McGonigle and Ruggeri 2014). Both animal and human exposure studies additionally suffer from ethical concerns, are cost-intensive and usually cannot be performed with the high number of individuals that would be necessary to overcome the problem of inter-individual variation.

In vitro systems still provide only a (usually very small) part of the whole organism, hence systemic reactions, the buffering capacity of the whole organism (for example the possibility to detoxify certain compounds via the bloodstream and the liver) cannot, or can only partly, be simulated (Forbes 2000; Kim et al. 2014; Bakand et al. 2005). Further, in vitro systems usually do not allow a one-to-one simulation of the normal, in vivo method of sample administration as, for instance, the dynamics of exhaust inhalation cannot be fully reproduced.

Nevertheless, in vitro models are a valuable and indispensable alternative to in vivo studies. Perhaps the greatest advantage of in vitro systems is their high potential for standardization, which provides reproducibility and hence comparability of results, even if obtained by different research groups. Furthermore, the high efficiency, low cost and the absence of ethical concerns make in vitro systems very attractive, particularly for screening approaches on the results of which further studies can be designed.

Already in the 1950s, in their book ‘The Principles of Humane Experimental Techniques’, Burch and Russel (1959) introduced the “three Rs” (Reduction in the number of test animals, Refinement of test-protocols in order to minimize suffering and Replacement of animal tests) and thereby introduced the concept of alternatives to whole-animal test systems. Even though the principles initially received little attention, with the growth of the animal welfare movement in the 1970s the three Rs became a widely accepted ethical framework for conducting scientific experiments using animals humanely. Their existence has made and continues to make a significant contribution to the development of in vitro test methods (Bakand et al. 2005). In the course of this change in paradigm, a number of cell lines have been developed as in vitro models of drug and toxicity screening (Allen et al. 2008), and with respect to inhalation toxicology, in particular cell lines and ex vivo models representative for the (animal and human) respiratory tract (Rothen-Rutishauser et al. 2008; Muhlfeld et al. 2008; Forbes 2000; Constant et al. 2014; Alfaro-Moreno et al. 2008; Blank et al. 2006; Rothen-Rutishauser et al. 2005; Steimer et al. 2005). These models are being continuously improved upon and validated and are increasingly able to overcome the limitations generally suffered by in vitro models. In addition, since they are nowadays commonly used in combination with sophisticated aerosol exposure systems that can cope with many of the aforementioned challenges of sample processing (Asimakopoulou et al. 2011; Muller et al. 2010; Paur et al. 2008; Lichtveld et al. 2012; Aufderheide et al. 2003; Le Prieur et al. 2000; Cheng et al. 2003; Knebel et al. 2002; Holder et al. 2007), in vitro test methods are a reliable and indispensable tool for toxicity testing of air pollutants.

## Conclusive remarks

While diesel engines offer great advantages—they are among the key technologies that make the modern world as we know it possible—they are also the source of highly potent air pollutants that affect the environment and public health on a large scale.

The great potency of diesel engine emissions as environmental toxicants is not simply the result of their chemical composition, but also relies on the structure–function relationships of the human respiratory tract and how the particulate fraction of diesel exhaust bypasses its defense mechanisms. Inhalable diesel exhaust particles are not efficiently filtered from the inhaled air, penetrate the respiratory tract deeply, deposit on or translocate across the lung epithelium and can evade otherwise efficient clearance mechanisms. By carrying condensed compounds of potentially high toxicity on their surfaces, these particles function like Trojan horses, allowing semi-volatile and nonvolatile chemicals access to organs, fluids and cellular compartments they could not reach without particles as carriers. The active surfaces of the highly biopersistent solid particle cores are also able to exert adverse effects on biofluids and cells for prolonged periods of time. The overall result is the induction of oxidative stress, pulmonary, systemic inflammation and, partly as a consequence of the latter two and partly via separate pathways, the damage of genomic DNA and thereby the potential formation of mutations and ultimately tumors.

This review has not covered the various efforts aimed at decreasing diesel exhaust toxicity by improved engine technologies and efficient exhaust after-treatment, as well as the ever-increasing stringency of exhaust emission legislation, which further accelerates the development of the latter technologies. The reductions in the emissions of toxic exhaust components achieved in recent years are impressive. Because of the high complexity of diesel engine emissions and the biological systems they interact with, it is unlikely that prediction of exhaust toxicity from exhaust composition will be possible in the near future, hence detailed toxicological studies will still be required. This need will become even more critical given the rapid changes in exhaust composition due to new technologies. Recent developments in the field of in vitro toxicology in combination with the availability of sophisticated aerosol exposure systems provides hope that even though exhaust toxicity will not be predictable in the near future, fast and reliable assessment of new technologies will be possible, and will allow exhaust emission legislation that effectively protects public health to be put in place.
